# An exploration of third parties’ preference for compensation over punishment: six experimental demonstrations

**DOI:** 10.1007/s11238-018-9665-9

**Published:** 2018-05-14

**Authors:** Janne van Doorn, Marcel Zeelenberg, Seger M. Breugelmans

**Affiliations:** 1 Department of Criminology, Leiden University, PO Box 9520, 2300 RA Leiden, The Netherlands; 2 Department of Social Psychology, Tilburg Institute for Behavior Economics Research, Tilburg University, PO Box 90153, 5000 LE Tilburg, The Netherlands; 3 VU Amsterdam, Amsterdam, The Netherlands

**Keywords:** Third party, Preference, Compensation, Punishment, Equity, Injustice

## Abstract

Research suggests that to restore equity, third parties prefer compensation of a victim over the punishment of a perpetrator. It remains unclear, however, whether this preference for compensation is stable or specific to certain situations. In six experimental studies, we find that adjustments in the characteristics of the situation or in the available behavioral options hardly modify the preference of compensation over punishment. This preference for compensation was found even in cases where punishment might refrain a perpetrator from acting unfairly again in the future, and even when punishment has a greater impact in restoring equity than compensation does. Thus, the preference of compensation over punishment appears to be quite robust. Implications and ideas for future research are discussed.

## Introduction

Injustice and unfairness are among the most agitating and frustrating experiences. We can experience injustice done to ourselves, but we can also observe injustice being done to someone else. Research shows that in cases where people receive an unjust treatment, they are motivated to punish the transgressor to restore justice (e.g., Fehr and Fischbacher 2004; Nelissen and Zeelenberg 2009; Seip et al. 2014). However, cases where they observe that someone else receives an unjust treatment, reactions to restore justice are more diverse (Gromet and Darley 2009). The current literature on reactions to injustice by third parties posits that there are two ways an observer might react: observers could either punish the perpetrator or compensate the victim (e.g., Adams and Mullen 2015; Chavez and Bicchieri 2013; Darley and Pittman 2003; FeldmanHall et al. 2014; Leliveld et al. 2012; Lotz et al. 2011; Van Doorn et al. 2014, 2017; Van Doorn et al. 2018; Van Prooijen 2010; Van de Vyver and Abrams 2015).

The previous theory and research on the third-party reactions has predominantly focused on punishment (e.g., Fehr and Fischbacher 2004; Goldberg et al. 1999; Henrich et al. 2006). Punishment is regarded as the intuitive human response to injustice (e.g., Carlsmith et al. 2002; Darley and Pittman 2003; Gromet and Darley 2009; Van Prooijen 2010). Less emphasis is put on compensation. Interestingly, the paper by Kahneman et al. (1986, p. S290), introducing the third-party punishment, actually designed their Experiment 2 “to establish whether people are willing to incur a cost to reward fairness and to punish unfairness when the fair or unfair actions were directed at someone else.” They offered participants to share money with two other participants, one who made a fair offer in a previous round and one who made an unfair offer. Participant could then choose between A) $5 dollar for oneself, $5 for the fair other and $0 for the unfair other, versus B) $6 dollar for oneself, $0 for the fair other, and $6 for the unfair other. Most participants (74%) chose to sacrifice $1 and thereby ‘reward’ the fair and ‘punish’ the unfair other. However, because, in this study, punishment and reward are perfectly correlated, we have no information about which tendency would be stronger. Turillo et al. (2002) disentangled both tendencies, finding support for the idea that self-sacrifice is stronger when rewarding fairness than when punishing unfairness. That is, decision-makers were more likely to split a relatively smaller sum ($5) with a stranger who had shown generous intent in the past than a larger amount ($6) with a stranger who had shown greedy intent in the past. Hence, research on reactions towards actors who could be fair or unfair strongly suggests that people care about fairness and that they are willing to make sacrifices to reward fairness. This also suggests that the contemporary focus on punishment in third-party reactions may only tell part of the story.

In this paper, we extend this idea to situations where people can choose to restore fairness by either punishing a perpetrator or compensating a victim. As stated before, most contemporary studies focus on punishing a perpetrator. However, restoring fairness by compensating the harm done to a victim may lead to additional beneficial outcomes such as increased interpersonal trust (e.g., Desmet et al. 2011), and cooperation (e.g., Fehr and Gächter 2002; Haesevoets et al. 2014). Therefore, what happens when people have to choose between punishment and compensation?

Recent studies including both punishment of a perpetrator and compensation of a victim as behavioral options reported that the third parties seem to prefer the latter option (Chavez and Bicchieri 2013; Leliveld et al. 2012); Lotz et al. 2011; Van Doorn et al. 2018). For example, in studies by Lotz et al. (2011) and Van Doorn et al. (2018), people observed a game played by two players (an allocator and a recipient) in which a certain amount of money needed to be divided. If allocators decided to allocate only a small amount of money to the other player, the third parties more often chose to spend their own money to compensate victims for their losses instead of punishing allocators for making the unfair distribution.

There is also research suggesting that people prefer punitive justice interventions to compensatory justice interventions (Adams and Mullen 2015; Van Prooijen 2010). However, in these cases, the option to punish or compensate did not occur at participants’ own expense. That is, participants did not have to sacrifice their own (hypothetical) money for compensation and punishment to occur. Instead, participants were asked to what extent they thought the perpetrator/victim should be punished/compensated using scale measures. In studies in which the behavioral options were costly, participants were often given the choice to spend (hypothetical) money or experimental currency which they received during the study on punishment and/or compensation. In such studies, compensation was preferred to punishment (Chavez and Bicchieri 2013; Leliveld et al. 2012; Lotz et al. 2011; Van Doorn et al. 2018). This raises the question as to why costliness would define the choice for a certain justice intervention.

When justice-restoring interventions are costly, it is very likely that observers will choose an intervention that is most beneficial to them. Punishment might elicit a retributive motivation by the perpetrator (possibly resulting in a spiral of aggression), whereas compensation allows for relationship building with the victim (e.g., Haesevoets et al. 2014; Okimoto and Tyler 2007). Compensation elicits a possibility of receiving social rewards (e.g., gain respect, approval of others, and recognition) and future reciprocal help (e.g., Lotz et al. 2011; Oarga et al. 2015; O’Gorman et al. 2005).

These findings made us wonder to what extent the preference for compensation would be generalizable across situations. In other words, how robust is this preference? We believe that it is important to answer this question, because it teaches us how people try to resolve situations of inequity or social transgressions. This, in turn, is important for contemporary models of human cooperation beyond kinship ties (e.g., Nowak 2006). The previous research has shown that human cooperation is best supported by repeated positive interactions with others (Rand et al. 2009). Furthermore, punishment has often been the only option given to participants, especially in the context of economic games, which has made punishment for the dominant response in cases of inequity, unfairness, or norm violations (e.g., Fehr and Fischbacher 2004; Goldberg et al. 1999; Nelissen and Zeelenberg 2009). Zhang and Ortmann (2016) already demonstrated that (context) differences in choice sets influence antisocial and prosocial decisions in a two-party setting. The previous research on the third-party punishment fuels the impression that people, in general, are very punitive. The current research sheds more light on how justified the punitive impression of third parties is.

In a broader sense, comparing preferences for punishments and for compensations can also provide valuable insights for the field of law. For example, if the general public prefers compensation of a victim over punishment of a perpetrator, while punishment is the dominant justice-restoring device in tort cases, this might damage public confidence in the criminal justice system and its ability to produce compliance (e.g., Bennett 2014; Tyler 1990; Robinson and Darley 1997). The influence of the general public’s opinion should not be underestimated. After all, punishments are financed with tax money, as well as some instances of compensation. Countries often have a so-called victim compensation fund (financed by tax money in the form of government grants), in which victims receive financial compensation when the perpetrator is not able to finance the damages. Hence, one could argue that punishment and compensation can be costly in real life, as well.

In the studies presented in this paper, we investigated the robustness of the preference for compensation over punishment in the third parties, by taking into account three important contextual factors: the notions of repeated unfairness, impact on fairness restoration, and self-other decision-making. With regard to the first factor of repeated unfairness, research has shown that deterrence of future offenses is one of the justifications for why people punish (see Carlsmith et al. 2002). When a perpetrator is repeatedly unfair, people are more inclined to punish or to advise harsher punishment (e.g., Roberts 1997; Seip et al. 2014). Based on these findings, we would hypothesize that people have a preference for punishment over compensation when a perpetrator is repeatedly unfair. The previous third-party compensation and punishment studies did not have the opportunity to find such an effect, because these studies were conducted in a nonrepeated setting (e.g., Chavez and Bicchieri 2013; Lotz et al. 2011; Van Doorn et al. 2018).

With regard to the impact or effectiveness of fairness restoration, people might prefer compensation over punishment, because punishment still leaves the victim in a disadvantageous position. In addition, people appear to be reluctant to do harm and rather not act negatively if they can avoid it (Baron 1995). In fact, some researchers have even argued that altruistic punishment is unlikely to occur at all (Pedersen et al. 2013). Punishment restores equity by lowering the position of the perpetrator, but does not restore the harm done to the victim (Darley and Pittman 2003). This could create a threshold for punishment, in the sense that people only consider it when its expected impact on the restoration of fairness is large enough compared to disutility of not compensating the victim. The previous research has not been able to test this option, because the monetary compensation and punishment were equally costly with an equal influence on the total amount of money victims and perpetrators owned (Chavez and Bicchieri 2013; Leliveld et al. 2012; Lotz et al. 2011; Van Doorn et al. 2018). In extension of this research and in line with the reasoning about punishment and compensation above, we hypothesize that people do prefer punishment over compensation (in a nonrepeated setting) when the punishment for the perpetrator is more beneficial for equity than the compensation for the victim.

With regard to self-other decision-making, people might be more inclined to choose punishment when the act is performed by someone else. Research on self-other decision-making has revealed that there is a difference in how individuals make a decision with a negative impact for themselves as compared to advising someone else about the decision. Giving advice about making a decision is more socially distant than making the decision yourself (Polman and Emich 2011) and is judged as being easier (Kray and Gonzalez 1999). This distance may subsequently lead to a more abstract perception and evaluation of the situation (Ledgerwood et al. 2010; Trope and Liberman 2010), making it easier for people to opt for the more straightforward option to punish, instead of evaluating other options such as compensation and its consequences. Finally, not bearing the responsibility of the decision oneself also means a lower risk of incurring potential reprisals from the perpetrator. We thus hypothesize that third parties are inclined to choose punishment when performed by someone else, as the ‘burden’ of punishment is also shifted to this other person.

Taken together, we present a series of six experiments to investigate the robustness of the preference for compensation over punishment. Specifically, we examined how robust this preference is in repeated interaction (Experiments 1–3), and when punishment has a greater impact than compensation (Experiment 4). In Experiment 5, we tested a methodological constraint, namely whether people increase punishment when compensation is not possible. In Experiment 6, we investigated whether people would shift to a preference for punishment when they do not have to do the punishment themselves. All studies also include the option to keep one’s endowment, making both punishment and compensation costly. This option has been included in the previous research, as well (e.g., Leliveld et al. 2012; Lotz et al. 2011; Van Doorn et al. 2018), and allows for investigating whether the potential absence of punitiveness is driven by people’s (prosocial) motivation to compensate or by people’s motivation not to act at all (as in daily life people generally have the option to not intervene as well). The basic finding in all six experiments is that participants generally prefer to compensate victims over punishing perpetrators.

## Experiment 1^1^

Three hundred and eight U.S. Amazon Mechanical Turk (“MTurk”) workers^2^ (170 males, 138 females; *M*_age_ = 31.86, SD = 10.62) were randomly assigned to the *single game* condition or *multiple games* condition. Participants in the single (multiple) game condition read the following scenario:

You observe a game (a series of games) played by Mark and Rick. In this (every) game, $100 needs to be divided. (In every game) Mark gets to decide how to divide this money between himself and Rick. Rick has no influence on the division of the money. (In the first game) Mark decides to give Rick $20 and to keep $80.

Next, we assessed our primary dependent variable, the choice for compensation, punishment, or keeping the money. This was done as follows in all studies (unless indicated differently). Participants read that they themselves owned $50, and that they could choose one of the three options to use the dollars they owned: They could compensate Rick; every dollar that they used for compensation would increase Rick’s amount with $3. They could punish Mark; every dollar that they used for punishment would decrease Mark’s amount with $3. Or they could keep the money themselves. When they choose to punish or to compensate, they also indicated how many dollars they would spend. We also included the option for participants to keep the money, to investigate whether the potential absence of punitiveness is driven by people’s (prosocial) motivation to compensate or by people’s motivation not to act at all (see Leliveld et al. 2012).

The choices are shown in Table 1. The conditions did not differ in choices, *χ*^2^ (2, *N * =308) = 1.33, *p * = 0.515. There were also no differences in the amount of money spent on compensation between the single game condition (*M * = $18.26, SD = 12.93) and the multiple game condition (*M* = $19.45, SD = 11.87), *t*(102) = − 0.49, *p* = 0.627. Participants in the single game condition, on average, spent $27.20 (SD = 19.25) on punishment; participants in the multiple games condition $20.11 (SD = 9.94). We could not compare the amount of money spent on punishment between conditions, and the amount of money spent on punishment and compensation within each condition, because of the low number of participants choosing punishment (Table 2).

**Table 1:** Choices for compensation, punishment, or keeping the money as a function of condition in Experiments 1–6

Choice	Exp. 1 *χ*^2^ (2, *N * =308) = 1.33, *p * = 0.515		Exp. 2 *χ*^2^ (2, *N * =137) = 1.64, *p * = 0.441		Exp. 3 *χ*^2^ (2, *N * =201) = 29.32, *p *< 0.001	
	Single game (*n* = 153) [%]	Multiple games (*n* = 155) [%]	Single game (*n* = 67) [%]	Multiple games (*n* = 70) [%]	Repeated unfairness (*n* = 100) [%]	Repeated fairness (*n* = 101) [%]
Compensation	31.4	37.4	56.7	52.9	39.0	22.8
Punishment	7.2	5.8	7.5	14.3	20.0	2.0
Keep	61.4	56.8	35.8	32.8	41.0	75.2

Choice	Exp. 4 *χ*^2^ (2, *N * =202) = 4.34, *p * = 0.114		Exp. 5 *χ*^2^ (2, *N * =204) = 49.83, *p *< 0.001		Exp. 6 *χ*^2^ (4, *N * =303) = 1.16, *p * = 0.885		
	Equal impact (*n* = 101) [%]	Unequal impact (*n* = 101) [%]	Two options (*n* = 102) [%]	Three options (*n* = 102) [%]	Third-party decision (*n* = 101) [%]	Fourth-party own decision (*n* = 101) [%]	Fourth-party other decision (*n* = 101) [%]
Compensation	41.6	27.7	–	37.3	38.8	34.6	39.6
Punishment	7.9	10.9	15.7	3.9	8.2	9.6	10.9
Keep	50.5	61.4	84.3	58.8	53.1	55.8	49.5

**Table 2:** Mean money spent (standard deviation in parentheses) on compensation and punishment as a function of condition in Experiments 1–6

Amount spent	Exp. 1		Exp. 2		Exp. 3	
	Single game	Multiple games	Single game	Multiple games	Repeated unfairness	Repeated fairness
Compensation	$18.26 (12.93)	$19.45 (11.87)	€18.50 (10.76)	€17.78 (10.21)	$28.87 (14.50)	$23.43 (15.95)
Punishment	$27.20 (19.25)	$20.11 (9.94)	€16.25 (7.50)	€20.25 (7.30)	$34.32 (16.28)	$10 (*n * = 1)

Choice	Exp. 4		Exp. 5		Exp. 6		
	Equal impact	Unequal impact	Two options	Three options	Third-party decision	Fourth-party own decision	Fourth-party other decision
Compensation	$17.94 (11.52)	$26.78 (14.35)	–	$15.33 (7.18)	$17.87 (9.94)	$22.19 (14.71)	$24.79 (15.35)
Punishment	$24.50 (10.85)	$22.91 (10.44)	$13.31 (6.62)	$15.50 (7.14)	$16.88 (5.30)	$12.78 (5.65)	$19.40 (12.38)

Participants more often chose for compensation than punishment, in both the single game condition, *χ*^2^ (1, *N * =59) = 23.20, *p *< 0.001, and the multiple game condition, *χ*^2^ (1, *N * =67) = 35.84, *p *< 0.001. Finally, participants more often chose for keeping the money themselves than for compensation, in both the single game condition, *χ*^2^ (1, *N * =142) = 14.90, *p *< 0.001, and the multiple game condition, *χ*^2^ (1, *N * =146) = 6.16, *p * = 0.013.

It thus appears that people are reluctant to punish when they have an opportunity to compensate a victim, even if a perpetrator might act unfairly again in the future. In the next study, adjusted versions of the scenarios used in Experiment 1 were used. It might not have been clear for participants how many games which Mark would play in the multiple games condition and that Mark could be repeatedly unfair. We also made it clear that it was a purely random choice to let Mark decide how to distribute the money.

## Experiment 2

One hundred and thirty-seven university students^3^ (25 males, 112 females; *M*_age_ = 19.44, SD = 2.39) were randomly assigned to the single game condition or multiple game condition and received course credit for their participation. Participants in the single (multiple) game condition read the following scenario and next indicated what to do with the $50 (compensate, punish, or keep):

You observe a single game (a series of 20 games) played by Mark and Rick in which (and in every game) €100 needs to be divided. By the toss of a coin, Mark is selected to decide how to divide this money between himself and Rick (in every game). Rick has no influence on the division of the money. (In the first game) Mark decides to give Rick €20 and to keep €80.

The choices are shown in Table 1. The conditions again did not differ, *χ*^2^ (2, *N * =137) = 1.64, *p * = 0.441. There were also no differences in the amount of money spent on compensation between the single game condition (*M * = €18.50, SD = 10.76) and the multiple game condition (*M* = €17.78, SD = 10.21), *t*(72) = 0.29, *p* = 0.770. Participants in the single game condition on average spent $16.25 (SD = 7.50) on punishment; participants in the multiple games condition $20.25 (SD = 7.30). We could not compare the amount of money spent on compensation with the amount of money spent on punishment, because of the low number of participants choosing punishment.

Participants more often choose for compensation than for punishment, in both the single game condition, *χ*^2^ (1, *N * =43) = 25.33, *p *< 0.001, and the multiple game condition, *χ*^2^ (1, *N * =47) = 15.51, *p *< 0.001. Finally, participants marginally significantly more often chose for compensation than for keeping the money themselves in the single game condition, *χ*^2^ (1, *N * =62) = 3.16, *p * = 0.075. The same pattern is found for participants in the multiple game condition, *χ*^2^ (1, *N * =60) = 3.27, *p * = 0.071.

From these results, we can conclude that when a perpetrator could be repeatedly unfair, people are still reluctant to punish and prefer compensation over punishment. However, from these results, we cannot determine whether participants choose to compensate the victim, because Mark could be unfair to this victim multiple times, making compensation for this particular victim the more ‘necessary’, or whether participants would also choose to compensate this victim if Mark had been unfair to multiple other victims. Hence, in the next experiment, we wanted to investigate whether people would still prefer compensation when we vary to whom the unfairness was done. We again adjusted the scenario, so that participants are confronted with Mark acting unfairly multiple times to multiple victims.

## Experiment 3

Two hundred and one U.S. MTurk-workers (112 males, 89 females; *M*_age_ = 33.65, SD = 11.01) were randomly assigned to the repeated unfairness condition or repeated fairness condition (acting as a control condition). Participants in the repeated unfairness condition read the following scenario:

You observe a series of 10 games played by Mark, and among others, Tim, Michael, and Rick. In every game, $100 needs to be divided. By means of a coin flip, it is decided that Mark gets to divide the money between himself and the other player. The other player has no influence on the division of the money. In the first game, Mark decides to give Tim $20 and to keep $80. In the second game, Mark decides to give Michael $10 and to keep $90. In the third game, Mark decides to give Rick $20 and to keep $80. Thus, Mark always keeps the greater part of the money and gives a small part to the other player.

Participants in the repeated fairness (control) condition read the same scenario, but here Mark divided the money between himself and the other players equally. Next, participants read that:

Shortly, Mark will play a fourth game with Daniel. You own $50 in this fourth game. How would you spend the $50 as an observer of this game? You have three options to spend each dollar: You can compensate Daniel; every dollar that you use for compensation will increase Daniel’s amount with $3. You can punish Mark; every dollar that you use for punishment will decrease Mark’s amount with $3. Or you can keep the money.

In the repeated unfairness condition, participants subsequently read: “In the fourth game, Mark decides to give Daniel $20 and to keep $80”. In the repeated fairness condition, participants read: “In the fourth game, Mark decides to give Daniel $50 and to keep $50”. Finally, participants were asked which option (punishment, compensation, or keeping the money) they would choose.

The choices are shown in Table 1. Participants in the repeated unfairness condition more often chose for punishment and compensation than participants in the repeated fairness condition, *χ*^2^ (2, *N * =201) = 29.32, *p *< 0.001, whereas participants in the latter condition more often chose to keep the money. There were again no differences in the amount of money spent on compensation between the repeated unfairness condition (*M * = $28.87, SD = 14.50) and the repeated fairness condition (*M* = $23.43, SD = 15.95), *t*(59) = 1.37, *p* = 0.177^4^. The amount of people choosing punishment within the repeated unfairness condition was considerably higher than in the previous two studies, and this group on average spent $34.32 (SD = 16.28) on punishment. Hence, we compared the amount of money spent on compensation and punishment for this group, but the difference was not significantly different, *t*(55) = − 1.28, *p* = 0.205.

Although the amount of money spent on compensation and punishment did not differ within the repeated unfairness condition, participants more often chose for compensation than for punishment within this condition, *χ*^2^ (1, *N * =59) = 6.12, *p * = 0.013. Participants in the repeated fairness condition more often chose for compensation over punishment, as well, *χ*^2^ (1, *N * =25) = 17.64, *p *< 0.001. Furthermore, within the repeated unfairness condition, the choice for compensation or keeping the money did not differ, *χ*^2^ (1, *N * =80) = 0.50, *p * = 0.823. Participants in the repeated fairness condition more often chose for keeping the money themselves, *χ*^2^ (1, *N * =99) = 28.37, *p *< 0.001.

The first series of experiments leads us to the preliminary conclusion that irrespective of whether a perpetrator can act unfairly again in the future or has been repeatedly unfair, people seem to prefer compensation over punishment. This finding might be surprising, as the compensation in the current experiment is given specifically to one victim, whereas the punishment could be viewed as given to all transgressions. Furthermore, punishment might be seen as a more viable option to deter the perpetrator from future offenses.

The preference for compensation might stem from the impression that the perpetrator is someone that will not change his behavior, because of the repeated unfairness already described in the scenario. Compensation has the sure benefit of restoring the harm done to the victim, as opposed to punishment in which the potential benefit of a perpetrator (or others in society) not committing any subsequent transgressions is more insecure. This could create a threshold in which people only consider punishment when its expected impact on the restoration of fairness is large enough compared to disutility of not compensating the victim. Hence, to make people choose for punishment, the benefit of this option should probably be greater than that of compensation. In Experiment 4, we, therefore, varied the impact of punishment and compensation in restoring injustice. Participants will read that the impact of compensation and punishment is equal or unequal. In the unequal condition, the impact of punishment is greater than compensation, which might motivate people to choose punishment over compensation.

## Experiment 4

Two hundred and two U.S. MTurk-workers (138 males, 64 females; *M*_age_ = 31.28, SD = 9.33) were randomly assigned to the equal impact condition or unequal impact condition. Participants read the scenario from the single game condition of Experiment 1. In the equal impact condition, the dependent variable was the same as in Experiment 1. In the unequal impact condition, the impact of punishment was greater than that of compensation: Every dollar used for punishment would decrease Mark’s amount with $3, while $1 compensation would increase Rick’s amount with $1.

The choices are shown in Table 1. There were no differences between conditions in monetary choice, *χ*^2^ (2, *N * =202) = 4.34, *p * = 0.114. When looking specifically at the choice to compensate or not, we do see that participants in the equal impact condition more often chose for compensation than participants in the unequal impact condition, *χ*^2^ (1, *N * =202) = 4.29, *p * = 0.038, but participants in the unequal impact condition (*M* = $26.78, SD = 14.35) spent more on compensation than participants in the equal impact condition (*M * = $17.94, SD = 11.52), *t*(67) = − 2.83, *p* = 0.006. Participants in the equal impact condition on average spent $24.50 (SD = 10.85) on punishment; participants in the unequal impact condition $22.91 (SD = 10.44). We could not compare the amount of money spent on compensation with the amount of money spent on punishment, because of the low number of participants choosing punishment.

Participants more often chose for compensation than for punishment in both the equal impact condition, *χ*^2^ (1, *N * =50) = 23.12, *p *< 0.001, and the unequal impact condition, *χ*^2^ (1, *N * =39) = 7.41, *p * = 0.006. In the unequal impact condition, participants more often chose for keeping the money themselves than for compensation, *χ*^2^ (1, *N * =90) = 12.84, *p *< 0.001. These findings indicate that when compensation has a low(er) impact, it is less preferred. In the equal impact condition, this difference was not significant, *χ*^2^ (1, *N * =93) = 0.87, *p * = 0.351.

In line with the previous experiments, people seem to prefer compensation over punishment, despite the fact that the impact of punishment was greater than that of compensation. We do find that when punishment has a greater impact, people less often choose for compensation. However, instead of seeing a larger total of people choosing for punishment, we see a larger total of people choosing to keep the money themselves. A potential explanation for this finding is that a confrontation with the perception that punishment has a greater impact than compensation might make people think that compensation is a ‘waste’ of their money. However, as the alternative of punishment is a response people generally do not want to opt for in the first place, they decide to keep the money themselves.

This latter finding made us wonder whether people would also opt for keeping the money if the option to compensate is not present at all, as in the case of the classic third-party punishment studies. Zhang and Ortmann (2016) have shown the importance of studying differences in choice sets to understand and predict pro- and antisocial decision-making. On the basis of previous third-party punishment studies, in which third parties were motivated to punish transgressors at the expense of their own resources (e.g., Fehr and Fischbacher 2004; Fehr and Gächter 2002; Henrich et al. 2006; Leibbrandt and López-Pérez 2012; Nelissen and Zeelenberg 2009), one would expect that participants are more inclined to punish when they have no option or knowledge of the option to compensate. However, if participants have an intrinsic preference for compensation, one would expect that they are more likely to not act at all if an option to compensate is not available in the first place. In Experiment 5, we explored participants’ choices when the option to compensate is absent.

## Experiment 5

Two hundred and four U.S. MTurk-workers (130 males, 74 females; *M*_age_ = 31.74, SD = 9.21) were randomly assigned to the two options (punishment/keeping the money) condition or three options (punishment/compensation/keeping the money) condition. Participants read the scenario from the single game condition of Experiment 1, and responded to the same dependent variable. The only difference was that, in the two-option condition, they could only choose between punishment and keeping the money.

The choices are shown in Table 1. Participants in the two-option condition more often chose to keep the money themselves than participants in the three-option condition, *χ*^2^ (2, *N * =204) = 49.83, *p *< 0.001. Participants in the two-option condition more often chose to keep the money than to punish. Participants in the three-option condition more often chose to compensate than to punish, *χ*^2^ (1, *N * =42) = 27.52, *p *< 0.001, and more often chose for keeping the money themselves than for compensation, *χ*^2^ (1, *N * =98) = 4.94, *p * = 0.026. This experiment thus again documents a preference for compensation over punishment. Participants in the two-option condition on average spent $13.31 (SD = 6.62) on punishment; participants in the three-option condition $15.50 (SD = 7.14). Because of the low number of people in the three-option condition choosing punishment, we could not test for statistical differences between these amounts.

As variations in the unfair situation and the spectrum of behavioral options did not seem to alter the preference for compensation, we sought to investigate whether it makes a difference *who* is distributing the punishment and compensation. That is, in the final experiment, we investigate whether people are less reluctant to punish if the act is performed by someone else. Therefore, in what follows either participants themselves were responsible for deciding how to spend the $50 or someone else was responsible for deciding how to spend the money.

## Experiment 6

Three hundred and three U.S. MTurk-workers (161 males, 142 females; *M*_age_ = 31.98, SD = 10.71) were randomly assigned to the third-party decision condition, fourth-party own decision or fourth-party other decision condition. Participants read the scenario from the single game condition of Experiment 1, with the small adjustment that participants in the third-party decision condition read that they observed this game between Mark and Rick and participants in the two-fourth-party conditions read that they and another person observed the game between Mark and Rick (and together owned $50). The dependent variable was again the choice for compensation, punishment, or keeping the money. In the fourth-party own decision condition, participants read that they were the person responsible for choosing. In the fourth-party other decision condition, participants read that the other person was responsible for choosing and they were asked how they would like to other person to spend the $50. Hence, the difference between conditions lies in (1) whether there is a fourth party, and (2) whether the participant (as a third party) or the fourth party is responsible for the decision.

The choices are shown in Table 1. The conditions did not differ, *χ*^2^ (4, *N * =303) = 1.16, *p * = 0.885. There were also no differences in the amount of money spent on compensation, *F*(2, 110) = 2.56, *p* = 0.082, *η*_p_^2^ = 0.044. Participants in the third-party decision condition (*M * = $17.87, SD = 9.94) spent an equal amount of money on compensation as those in the fourth-party own decision condition (*M * = $22.19, SD = 14.71), *p* = 0.358, and the fourth-party other decision condition (*M* = $24.79, SD = 15.35), *p* = 0.068. Participants in the third-party decision condition on average spent $16.88 (SD = 5.30) on punishment; participants in the fourth-party own decision condition $12.78 (SD = 5.65); and participants in the fourth-party other decision condition $19.40 (SD = 12.83). We could not compare the amount of money spent on compensation with the amount of money spent on punishment because of the low number of participants choosing punishment.

Participants chose more often for compensation than for punishment in the third-party decision condition, *χ*^2^ (1, *N * =46) = 19.57, *p *< 0.001, in the fourth-party own decision condition, *χ*^2^ (1, *N * =46) = 14.70, *p *< 0.001, and in the fourth-party other decision condition, *χ*^2^ (1, *N * =51) = 16.49, *p *< 0.001. Finally, the choice for compensation or keeping the money did not differ within the third-party decision condition, *χ*^2^ (1, *N * =90) = 2.18, *p * = 0.140, and within the fourth-party other decision condition, *χ*^2^ (1, *N * =90) = 1.11, *p * = 0.292. However, within the fourth-party own decision condition participants more often chose for keeping the money, *χ*^2^ (1, *N * =94) = 5.15, *p * = 0.023. From this experiment, it can be concluded that irrespective of whether people are responsible for punishment or compensation themselves, people are not more inclined to punish.

## General discussion

The previous research seemed to indicate that third parties prefer compensation of a victim over punishment of a perpetrator in cases of unfair distributions of money (e.g., Chavez and Bicchieri 2013; Leliveld et al. 2012; Lotz et al. 2011; Van Doorn et al. 2018). In six experiments, we investigated the robustness of this preference for compensation over punishment in third parties. We hypothesized that third parties would be more inclined to prefer punishment over compensation when (1) a perpetrator could be repeatedly unfair, (2) the impact of punishment in restoring equity between perpetrator and victim was greater than the impact of compensation, and (3) a fourth party was responsible for making the (punitive) decision. These hypotheses were not confirmed. The preference for compensation was found to be very robust.

Experiments 1–3 showed that when a perpetrator can be or is unfair repeatedly to one or multiple victims, third parties still preferred compensation of a victim over punishment of the perpetrator. Experiment 4 showed that third parties prefer compensation over punishment irrespective of the impact of punishment in restoring equity being greater than the impact of compensation. Experiment 5 showed that when the option to compensate was absent, the majority chose not to intervene. Finally, in Experiment 6, we found that third parties preferred compensation over punishment when the act was performed by themselves as well as by others.

Across all studies, we found a very consistent pattern of results: compensation was preferred to punishment. We even found a very similar pattern in the percentages preferring compensation, punishment, or keeping the money in the baseline conditions of all studies (in these baseline conditions, one victim received an unequal amount of money from a dictator and participants had the option to compensate, punish, or keep the money^5^). Considering the amount of money spent to compensation and punishment, results are consistent with the choices participants make: when there is no difference in choosing compensation between conditions, there is also no difference in the amount of money spent on compensation. There was often no possibility to compare the amount of money spent on compensation with the amount of money spent on punishment in our studies because of the low number of participants actually choosing punishment.

The current studies aimed to test whether the preference for compensation over punishment generalizes across different situations, which it does. An interesting follow-up question would be why this preference for compensation exists. One answer could be that despite the increased impact of punishment in Experiment 4, and not that of compensation, the victim is still in a disadvantageous position and people want to see that restored. People might judge punishment as less effective, because punishment only makes sense if there is a good-enough probability that the behavior of the perpetrator will change. Compensation has the sure benefit of restoring the harm done to the victim; as opposed to punishment in which the potential benefit of a perpetrator (or others in society) not committing any subsequent transgressions is more insecure. Related to that, people might be more reluctant to punish than compensate, because, in economic games, punishment both costs the punisher and destroys value of the punished. Compensation on the other hand could be considered more of a transfer of money in which the victim gains money.

Furthermore, although it has not been explicitly mentioned that the participant would engage in future contact with the victim or perpetrator, it might be the case that compensation is preferred because of (implicit) relational concerns (Desmet and Leunissen 2014; Haesevoets et al. 2014; Okimoto and Tyler 2007), and/or a fear for retribution by the punished perpetrator. For example, O’Gorman et al. (2005) found that people were more willing to help a victim when they were told that there was a high possibility of future interaction with that victim as opposed to a low possibility of future interaction (see also Roberts et al. 2013). Compensation allows for investing in a relationship and positive reciprocity, whereas punishment does not. That is, compensating or helping a victim might elicit a reciprocal prosocial action from that victim and thus allows for relationship building, whereas punishment is not likely to lead to a reciprocal prosocial action by the perpetrator but might instead elicit an antisocial action in the form of reprisals.

Finally, it might also be the case that the unjust situations used in the current studies are not severe enough to elicit a preference for punitive responses. For example, Rucker et al. (2004) showed that the amount of punishment observers prescribed was higher in the case of a severe crime (carjacking resulting in the death of the victim) than in the case of a moderately severe crime (carjacking with only material harm to the victim). Although compensation is not possible in the aforementioned example of a severe crime, it might still be the case that, for severe crimes, people rely more on retributive motives than for less severe crimes when determining whether to punish or compensate (Gromet and Darley 2009). Hough and Roberts (1999) also illustrate that the general public is mostly punitive when it comes to the worst types of violent crime. Future research might be able to disentangle whether crime severity can explain the preference for compensation or punishment.

More generally, and related to the notion of severity, it is important to investigate whether this preference for compensation also generalizes to different ‘forms’ of unfairness besides an unequal distribution of money as used in all our studies. It might, for example, be interesting to see whether a preference for compensating a victim is also present in the case of assaults or robbery. However, as the aim of our studies was to test the robustness of the preference for compensation, the consistency in the dictator game design of our studies was essential.

Although there is a preference for compensation over punishment in our studies, we do not argue that people always prefer compensation over punishment. In fact, the percentage of people choosing to punish did increase in some situations. For example, in Experiment 3, people more often chose to punish when they were confronted with a perpetrator that had acted unfairly repeatedly as compared to a perpetrator that had acted fairly repeatedly. In addition, in Experiment 5, we see an increase in punishment when the option to compensate is not given. In Experiment 4, we find that, when punishment has a greater impact, people less often choose for compensation. However, instead of seeing a larger total of people choosing for punishment, we see a larger total of people choosing to keep the money themselves. We also see a surprisingly large number of participants choosing to compensate in the repeated fairness condition of Experiment 3, where there is not really a victim. It might be the case that people chose this prosocial option as it can be interpreted more as giving instead of compensating, and people derive pleasure from that. Furthermore, people might be reluctant to act negatively by taking away money or to be egoistic by keeping the money in a fair situation. Finally, if participants would be confronted with repeated unfairness more directly (for example, by specifically observing all the games in which different victims are involved), they might be more inclined to choose punishment over compensation.

Our studies also included the option not to act (keeping the money). When comparing the prosocial act with not acting at all results do not indicate a clear preference. Deciding to keep the money might be viewed as the self-interested option. However, in addition to that, it might be the case that people chose to not act, because they judge their act as interfering between other people’s business. This might especially be the case in situations that involve people that the participants were not acquainted with. In the case of friends or family, people might be more inclined to act (in a prosocial or antagonistic way) than not to act. For example, research has illustrated that people show more prosocial behavior when a victim is a (genetically or emotionally) close other or when feelings of distance between victims and potential benefactors are reduced (e.g., Loewenstein and Small 2007; Van Prooijen 2010).

We realize that our studies do not comprise actual behavior but hypothetical situations. Still, we believe that these studies are important as they are an initial test of the robustness of the preference for compensation over punishment. Furthermore, the choice for punishment in our ‘imaginary’ studies might even be easier than in studies where real choices are made and real costs are involved. Without real costs involved in punishment in our studies, we still find the robust preference for compensation over punishment. Hence, the preference which we find in our studies will most likely be equally if not more strongly present in actual behavior (see also Chavez and Bicchieri 2013; Van Doorn et al. 2018).

Our findings have implications in the domain of punishment. The previous research, especially in the context of economic games, often only included the option to punish which has made punishment the dominant response in cases of inequity, unfairness or norm violations (e.g., Fehr and Fischbacher 2004; Goldberg et al. 1999; Nelissen and Zeelenberg 2009; Seip et al. 2009, 2014). For example, Van Prooijen and Kerpershoek (2013) found that participants with an unfulfilled basic psychological need for autonomy showed increased punishment intentions towards a perpetrator, and explained these results by arguing that their autonomy-deprived participants had an increased concern for justice. However, the need for autonomy might have also been fulfilled by compensatory actions, as the concern for justice is related to compensation, as well (Lotz et al. 2013; Stavrova and Schlösser 2015). Hence, it is not necessarily the case which the autonomy-deprived people are more punitive; they might be more motivated to restore justice in general.

Finally, these results also have implications for justice and law. From these results, it appears that third parties prefer compensation of a victim over punishment of a perpetrator, while punishment is the dominant justice-restoring device in tort cases. This preference also holds when third parties do not have to indicate a preference themselves, but have to indicate how they would prefer someone to choose, which is similar to a third party advising a judge in dealing with injustice. If punishment is not the preferred manner in restoring equity or justice, a (severe) punishment might never match up to the impact that compensation establishes in observers’ eyes.

Taken together, the results show that third parties have a strong preference for compensation over punishment to restore equity. Although, in some situations, the percentage of people that want to punish the perpetrator does increase, the preference for compensation of victims seems to be a strong one. Even when punishment might refrain a perpetrator from acting unfairly again in the future, and even when punishment has a greater impact in restoring equity than compensation does, the preference for compensation remains. These findings imply that third parties prefer a prosocial option over an antagonistic option which has implications for theories on punishment, justice, and law.

## Footnotes

1 We report all conditions, measures, and data exclusions (if any) in all studies. There were no gender effects on the monetary choice (compensation; punishment; no act) in any of the studies. When there was an inequality of variances when testing differences between means using *t* tests, corrected degrees of freedom are reported throughout the article. All MTurk-workers had an HIT approval of 95% or more, and received $0.10–$0.25 for their participation. In all six experiments, we asked participants to indicate how much anger, shame, regret, pride, and guilt which they would feel in the described situation. These measures were included for exploratory reasons, and are not described in this article.

2 The use of Internet samples has become a common practice in behavioral science research. Importantly, it has repeatedly been demonstrated that MTurk participants as reliable as lab participants while providing greater diversity (e.g., Paolacci et al. 2010; Paolacci and Chandler 2014).

3 An additional three participants were excluded from the analyses, because they failed to follow the instructions.

4 There were two participants in the repeated fairness condition who chose to punish. However, only one participant reported the amount used for punishment which was $10.

5 Please note that these conditions might have been labeled differently depending on the specific design of the study, even though they are identical in nature.
